# *N*′-*tert*-Butyl-*N*′-(3,5-dimethyl­benzo­yl)-2,2-dimethyl-4-oxochroman-6-carbo­hydrazide

**DOI:** 10.1107/S1600536809028815

**Published:** 2009-07-29

**Authors:** Zhongzhen Zhou, Wenwei You, Zhongkun Tang, Peiliang Zhao

**Affiliations:** a School of Pharmaceutical Sciences, Southern Medical University, Guangzhou 510515, People’s Republic of China

## Abstract

In the crystal structure of the title compound, C_25_H_30_N_2_O_4_, the steric size of the *tert*-butyl group causes the 3,5-dimethyl­phenyl ring to adopt a *transoid* geometry with respect to the N—C(O) bond. The six-membered heterocyclic ring is disordered over two sites, with occupancies of 0.553 (4) and 0.447 (4). Intra­molecular C—H⋯O inter­actions are present. In the crystal, mol­ecules are linked by inter­molecular N—H⋯O and C—H⋯O hydrogen bonds.

## Related literature

For general background to dibenzoyl­hydrazines and their derivatives, see: Sawada *et al.* (2003 ▶). For a related structure, see: Zhao *et al.* (2005 ▶). For the preparation of the title compound, see: Zhao *et al.* (2007 ▶); Mao *et al.* (2008 ▶).
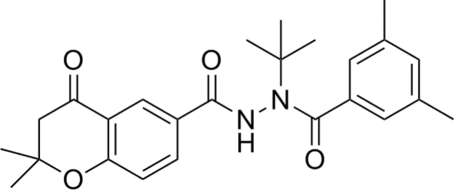


## Experimental

### 

#### Crystal data

C_25_H_30_N_2_O_4_
*M* *_r_* = 422.51
Monoclinic, 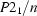

*a* = 14.1260 (12) Å
*b* = 10.6964 (9) Å
*c* = 15.4370 (13) Å
β = 96.426 (2)°
*V* = 2317.8 (3) Å^3^
*Z* = 4
Mo *K*α radiation
μ = 0.08 mm^−1^
*T* = 297 K
0.30 × 0.20 × 0.20 mm

#### Data collection

Bruker SMART APEX CCD area-detector diffractometer
Absorption correction: multi-scan (*SADABS*; Sheldrick, 2005 ▶) *T*_min_ = 0.976, *T*_max_ = 0.984
13199 measured reflections
5043 independent reflections
3983 reflections with *I* > 2σ(*I*)
*R*_int_ = 0.020

#### Refinement

*R*[*F* ^2^ > 2σ(*F* ^2^)] = 0.048
*wR*(*F* ^2^) = 0.142
*S* = 1.04
5043 reflections
338 parameters
H atoms treated by a mixture of independent and constrained refinement
Δρ_max_ = 0.22 e Å^−3^
Δρ_min_ = −0.17 e Å^−3^

### 

Data collection: *SMART* (Bruker, 2001 ▶); cell refinement: *SAINT* (Bruker, 2001 ▶); data reduction: *SAINT*; program(s) used to solve structure: *SHELXS97* (Sheldrick, 2008 ▶); program(s) used to refine structure: *SHELXL97* (Sheldrick, 2008 ▶); molecular graphics: *SHELXTL* (Sheldrick, 2008 ▶); software used to prepare material for publication: *SHELXTL*.

## Supplementary Material

Crystal structure: contains datablocks global, I. DOI: 10.1107/S1600536809028815/fj2236sup1.cif

Structure factors: contains datablocks I. DOI: 10.1107/S1600536809028815/fj2236Isup2.hkl

Additional supplementary materials:  crystallographic information; 3D view; checkCIF report

## Figures and Tables

**Table 1:** Hydrogen-bond geometry (Å, °)

*D*—H⋯*A*	*D*—H	H⋯*A*	*D*⋯*A*	*D*—H⋯*A*
C16—H16*C*⋯O4	0.96	2.39	3.007 (2)	121
C15—H15*A*⋯O4	0.96	2.36	2.918 (2)	117
C8—H8⋯O4^i^	0.93	2.48	3.3214 (18)	151
N1—H1⋯O4^i^	0.862 (17)	2.096 (18)	2.9442 (15)	167.8 (15)

Symmetry code: (i) 
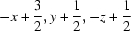
.

**Fig. 1.**
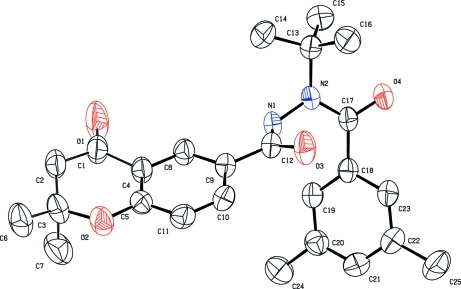
A view of the molecule of (I) showing the atom-labelling scheme. Displacement ellipsoids are drawn at the 50% probability level. H atoms are represented by circles of arbitrary size.

**Fig. 2.**
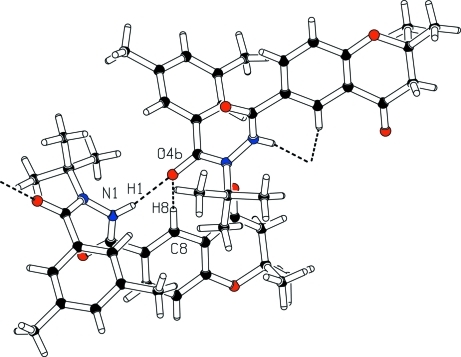
Hydrogen bonding in the crystal structure of (I).Hydrogen bonds are shown as dashed lines. [Symmetry codes: b. -x + 3/2,y + 1/2, -z + 1/2]

## supplementary crystallographic information

### Comment

Dibenzoylhydrazines are well known as nonsteroidal ecdysone agonists that have the potential to control the Lepidopteran pests while exerting only a low toxicity against non-target insects. (Sawada *et al.*, 2003). While chroman derivatives also have broad-spectrum biological activity. Hence, to search for novel lead compounds for crop protection, The title compound, (I), was designed and synthesized in our laboratory. In this paper, we present the X-ray crystallographic analysis of (I).

As shown in Fig. 1, the six-membered heterocyclic ring adopts a half-chair conformation. the dihedral angle between the phenyl ring and the benzene ring of the chromanone is 58.14 (2)°. The six-memebered heterocyclic ring is disordered over two sites with occupancies of 0.553 (4) and 0.447 (4). The steric size of the *N*-*tert*-butyl group causes the 3,5-dimethylphenyl group to be directed away from it.

One intermolecular N—H···O hydrogen bond and one intermolecular C—H···O hydrogen bonds exist in the crystal structure (Table 1 and Fig. 2), atoms N1 and C8 in the molecule act as donors, *via* the H atoms H1 and H8, to the O4 of a adjacent molecule(Tabel 2). As a result, an seven-membered ring is formed between molecules (Fig. 2). No π-π stacking interactions are observed in the crystal structure.

### Experimental

To A solution of 2,2-Dimethyl-4-oxo-chroman-6-carboxylic acid *N*'-*tert*-butyl-hydrazide (1.5 mmol) in 10 ml of dichloromethane was added dropwise to a stirred mixture of 3,5-dimethylbenzoyl chloride (1.5 mmol), triethylamine (1.6 mmol) and dichloromethane (5 ml) in an ice bath.After stirring the mixture at room temperature for 3 h,ethyl acetate (30 ml) was added to the reaction mixture. The organic layer was separated and washed successively with water (15 ml) and brine (15 ml), and then dried with anhydrous sodium sulfate. After evaporating the solvent, the residue was purified by column chromatography on silicagel using hexane/ethyl acetate (9:1 *v*/*v*) as eluent to afford (I) (yield 80%, m.p. 435 K) Spectroscopic analysis: ^1^H NMR(CDCl3, 400 MHz) 8.06 (s, 1H, N—H), 7.74 (d, 2H, C10—H, C11—H), 7.05 (s, 2H, C19—H, C23—H), 6.81 (m, 2H, C8—H, C21—H), 2.70 (s, 2H, C2—H), 2.21 (s, 6H, C24—H, C25—H), 1.57 (s, 9H, C14—H, C15—H,C16—H),1.44 (s, 6H, C6—H,C7—H); MS (EI 70 eV)m/*z*(%):422 (16), 367 (95), 349 (35), 203 (62), 146 (20),133 (100), 105 (49),102 (19). Crystals suitable for an X-ray diffraction study were grown from methanol at 292 K.

### Refinement

All H atoms were initially located in a difference Fourier map. The methyl H atoms were then constrained to an ideal geometry with C—H distances of 0.96 Å and *U*_iso_(H) = 1.5*U*_eq_(C), but each group was allowed to rotate freely about its C—C bond. All other H atoms were placed in geometrically idealized positions and constrained to ride on their parent atoms with C—H distances in the range 0.93–0.97 Å,an N—H distance of 0.86 Å and *U*_iso_(H) = 1.2*U*_eq_(C,*N*).

### Crystal data

**Table:**

C_25_H_30_N_2_O_4_	*F*(000) = 904
*M**_r_* = 422.51	*D*_x_ = 1.211 Mg m^−^^3^
Monoclinic, *P*2_1_/*n*	Mo *K*α radiation, λ = 0.71073 Å
Hall symbol: -P 2yn	Cell parameters from 5293 reflections
*a* = 14.1260 (12) Å	θ = 2.3–27.9°
*b* = 10.6964 (9) Å	µ = 0.08 mm^−^^1^
*c* = 15.4370 (13) Å	*T* = 297 K
β = 96.426 (2)°	Block, colorless
*V* = 2317.8 (3) Å^3^	0.30 × 0.20 × 0.20 mm
*Z* = 4	

### Data collection

**Table:**

Bruker SMART APEX CCD area-detector diffractometer	5043 independent reflections
Radiation source: fine-focus sealed tube	3983 reflections with *I* > 2σ(*I*)
graphite	*R*_int_ = 0.020
φ and ω scans	θ_max_ = 27.0°, θ_min_ = 2.1°
Absorption correction: multi-scan (*SADABS*; Sheldrick, 2005)	*h* = −18→18
*T*_min_ = 0.976, *T*_max_ = 0.984	*k* = −13→13
13199 measured reflections	*l* = −19→11

### Refinement

**Table:**

Refinement on *F*^2^	Primary atom site location: structure-invariant direct methods
Least-squares matrix: full	Secondary atom site location: difference Fourier map
*R*[*F*^2^ > 2σ(*F*^2^)] = 0.048	Hydrogen site location: inferred from neighbouring sites
*wR*(*F*^2^) = 0.142	H atoms treated by a mixture of independent and constrained refinement
*S* = 1.04	*w* = 1/[σ^2^(*F*_o_^2^) + (0.0739*P*)^2^ + 0.3537*P*] where *P* = (*F*_o_^2^ + 2*F*_c_^2^)/3
5043 reflections	(Δ/σ)_max_ = 0.027
338 parameters	Δρ_max_ = 0.22 e Å^−^^3^
0 restraints	Δρ_min_ = −0.17 e Å^−^^3^

### Special details

**Table:**

Geometry. All e.s.d.'s (except the e.s.d. in the dihedral angle between two l.s. planes) are estimated using the full covariance matrix. The cell e.s.d.'s are taken into account individually in the estimation of e.s.d.'s in distances, angles and torsion angles; correlations between e.s.d.'s in cell parameters are only used when they are defined by crystal symmetry. An approximate (isotropic) treatment of cell e.s.d.'s is used for estimating e.s.d.'s involving l.s. planes.
Refinement. Refinement of *F*^2^ against ALL reflections. The weighted *R*-factor *wR* and goodness of fit *S* are based on *F*^2^, conventional *R*-factors *R* are based on *F*, with *F* set to zero for negative *F*^2^. The threshold expression of *F*^2^ > σ(*F*^2^) is used only for calculating *R*-factors(gt) *etc*. and is not relevant to the choice of reflections for refinement. *R*-factors based on *F*^2^ are statistically about twice as large as those based on *F*, and *R*- factors based on ALL data will be even larger.

### Fractional atomic coordinates and isotropic or equivalent isotropic displacement parameters (Å^2^)

**Table:**

	*x*	*y*	*z*	*U*_iso_*/*U*_eq_	Occ. (<1)
C3	0.66849 (13)	1.10349 (14)	−0.10449 (11)	0.0605 (4)	
C4	0.67622 (12)	0.88189 (13)	−0.00473 (10)	0.0514 (4)	
C5	0.68639 (11)	0.87969 (14)	−0.09323 (9)	0.0494 (3)	
C1	0.6450 (5)	0.9997 (11)	0.0354 (8)	0.065 (2)	0.553 (4)
O1	0.6346 (7)	1.0033 (10)	0.1119 (7)	0.114 (3)	0.553 (4)
C2	0.6193 (3)	1.1038 (3)	−0.0289 (2)	0.0658 (10)	0.553 (4)
H2A	0.5515	1.0993	−0.0475	0.079*	0.553 (4)
H2B	0.6314	1.1830	0.0009	0.079*	0.553 (4)
C6	0.6317 (4)	1.1921 (4)	−0.1762 (3)	0.0794 (12)	0.553 (4)
H6A	0.5657	1.1746	−0.1940	0.119*	0.553 (4)
H6B	0.6676	1.1816	−0.2249	0.119*	0.553 (4)
H6C	0.6382	1.2765	−0.1553	0.119*	0.553 (4)
C7	0.7797 (3)	1.1317 (4)	−0.0800 (3)	0.0873 (13)	0.553 (4)
H7A	0.7875	1.2129	−0.0537	0.131*	0.553 (4)
H7B	0.8112	1.1295	−0.1319	0.131*	0.553 (4)
H7C	0.8069	1.0696	−0.0398	0.131*	0.553 (4)
C1'	0.6818 (6)	1.0036 (14)	0.0428 (10)	0.060 (2)	0.447 (4)
O1'	0.6711 (7)	1.0167 (11)	0.1174 (8)	0.0697 (17)	0.447 (4)
C2'	0.7037 (4)	1.1160 (4)	−0.0090 (3)	0.0685 (13)	0.447 (4)
H2'1	0.7721	1.1291	−0.0027	0.082*	0.447 (4)
H2'2	0.6746	1.1890	0.0143	0.082*	0.447 (4)
C7'	0.5527 (3)	1.0980 (4)	−0.1099 (3)	0.0755 (13)	0.447 (4)
H7'1	0.5340	1.0260	−0.0789	0.113*	0.447 (4)
H7'2	0.5262	1.0926	−0.1698	0.113*	0.447 (4)
H7'3	0.5297	1.1722	−0.0843	0.113*	0.447 (4)
C6'	0.6941 (6)	1.2082 (6)	−0.1583 (4)	0.0936 (19)	0.447 (4)
H6'1	0.6725	1.2850	−0.1350	0.140*	0.447 (4)
H6'2	0.6645	1.1974	−0.2169	0.140*	0.447 (4)
H6'3	0.7620	1.2110	−0.1583	0.140*	0.447 (4)
C8	0.68071 (12)	0.77025 (13)	0.04199 (10)	0.0515 (4)	
H8	0.6738	0.7719	0.1012	0.062*	
C9	0.69514 (10)	0.65732 (12)	0.00237 (9)	0.0440 (3)	
C10	0.70566 (10)	0.65759 (14)	−0.08622 (9)	0.0499 (3)	
H10	0.7153	0.5823	−0.1141	0.060*	
C11	0.70207 (11)	0.76656 (15)	−0.13332 (10)	0.0544 (4)	
H11	0.7102	0.7646	−0.1923	0.065*	
C12	0.69536 (10)	0.53436 (12)	0.04860 (10)	0.0468 (3)	
C13	0.59930 (11)	0.41391 (14)	0.21344 (11)	0.0538 (4)	
C14	0.53721 (13)	0.52843 (17)	0.18794 (15)	0.0753 (5)	
H14A	0.5673	0.6019	0.2140	0.113*	
H14B	0.4759	0.5178	0.2082	0.113*	
H14C	0.5296	0.5373	0.1257	0.113*	
C15	0.60593 (14)	0.39710 (18)	0.31198 (13)	0.0715 (5)	
H15A	0.6370	0.3193	0.3279	0.107*	
H15B	0.5430	0.3967	0.3299	0.107*	
H15C	0.6420	0.4647	0.3401	0.107*	
C16	0.55686 (12)	0.29932 (16)	0.16431 (14)	0.0702 (5)	
H16A	0.5496	0.3161	0.1028	0.105*	
H16B	0.4957	0.2808	0.1828	0.105*	
H16C	0.5985	0.2291	0.1764	0.105*	
C17	0.76979 (10)	0.35224 (11)	0.20322 (9)	0.0419 (3)	
C18	0.86257 (9)	0.37282 (12)	0.16645 (9)	0.0426 (3)	
C19	0.91465 (10)	0.48329 (13)	0.17253 (10)	0.0475 (3)	
H19	0.8915	0.5525	0.2000	0.057*	
C20	1.00090 (11)	0.49090 (14)	0.13791 (10)	0.0530 (4)	
C21	1.03369 (11)	0.38643 (16)	0.09734 (11)	0.0582 (4)	
H21	1.0906	0.3920	0.0725	0.070*	
C22	0.98488 (11)	0.27454 (15)	0.09244 (11)	0.0561 (4)	
C23	0.89924 (10)	0.26895 (13)	0.12762 (10)	0.0494 (3)	
H23	0.8655	0.1942	0.1253	0.059*	
C24	1.05961 (13)	0.60902 (17)	0.14489 (14)	0.0745 (5)	
H24A	1.0845	0.6224	0.2046	0.112*	
H24B	1.0203	0.6786	0.1246	0.112*	
H24C	1.1113	0.6011	0.1099	0.112*	
C25	1.02483 (16)	0.16056 (19)	0.05138 (16)	0.0862 (6)	
H25A	1.0685	0.1864	0.0114	0.129*	
H25B	0.9737	0.1137	0.0206	0.129*	
H25C	1.0577	0.1092	0.0961	0.129*	
N1	0.71169 (8)	0.54096 (10)	0.13664 (8)	0.0442 (3)	
H1	0.7207 (11)	0.6105 (16)	0.1645 (11)	0.053*	
N2	0.69837 (8)	0.43704 (10)	0.18823 (8)	0.0448 (3)	
O2	0.68091 (10)	0.98341 (11)	−0.14408 (8)	0.0697 (4)	
O3	0.68252 (10)	0.43588 (10)	0.00964 (8)	0.0680 (3)	
O4	0.75822 (7)	0.25569 (9)	0.24413 (7)	0.0527 (3)	

### Atomic displacement parameters (Å^2^)

**Table:**

	*U*^11^	*U*^22^	*U*^33^	*U*^12^	*U*^13^	*U*^23^
C3	0.0823 (11)	0.0413 (8)	0.0569 (9)	−0.0035 (7)	0.0036 (8)	0.0097 (7)
C4	0.0704 (9)	0.0384 (7)	0.0462 (8)	0.0063 (6)	0.0101 (7)	0.0034 (6)
C5	0.0560 (8)	0.0456 (8)	0.0478 (8)	0.0026 (6)	0.0105 (6)	0.0064 (6)
C1	0.100 (5)	0.040 (2)	0.059 (4)	0.013 (4)	0.027 (5)	0.004 (2)
O1	0.236 (10)	0.046 (3)	0.070 (4)	0.024 (5)	0.064 (6)	0.004 (2)
C2	0.092 (3)	0.0395 (14)	0.069 (2)	0.0125 (15)	0.0221 (18)	0.0105 (13)
C6	0.114 (4)	0.053 (2)	0.070 (2)	0.005 (2)	0.007 (3)	0.0194 (18)
C7	0.080 (2)	0.066 (2)	0.115 (3)	−0.0203 (18)	0.006 (2)	0.008 (2)
C1'	0.088 (6)	0.043 (3)	0.049 (3)	0.014 (5)	0.007 (5)	0.0066 (19)
O1'	0.121 (4)	0.042 (3)	0.045 (2)	0.010 (2)	0.008 (3)	−0.0031 (18)
C2'	0.090 (3)	0.047 (2)	0.066 (2)	−0.0059 (19)	−0.003 (2)	−0.0011 (17)
C7'	0.073 (3)	0.078 (3)	0.074 (3)	0.015 (2)	0.002 (2)	0.008 (2)
C6'	0.138 (6)	0.068 (3)	0.079 (4)	−0.023 (4)	0.030 (4)	0.018 (3)
C8	0.0718 (10)	0.0418 (7)	0.0416 (7)	0.0084 (7)	0.0097 (7)	0.0023 (6)
C9	0.0468 (7)	0.0375 (7)	0.0467 (7)	0.0039 (5)	0.0010 (6)	0.0006 (6)
C10	0.0540 (8)	0.0450 (8)	0.0503 (8)	0.0081 (6)	0.0043 (6)	−0.0066 (6)
C11	0.0645 (9)	0.0576 (9)	0.0427 (8)	0.0072 (7)	0.0129 (7)	0.0002 (7)
C12	0.0518 (8)	0.0355 (7)	0.0519 (8)	0.0041 (6)	0.0012 (6)	−0.0016 (6)
C13	0.0501 (8)	0.0415 (7)	0.0715 (10)	0.0016 (6)	0.0140 (7)	−0.0001 (7)
C14	0.0567 (10)	0.0608 (10)	0.1105 (16)	0.0147 (8)	0.0189 (10)	0.0057 (10)
C15	0.0796 (12)	0.0646 (10)	0.0756 (12)	0.0033 (9)	0.0318 (9)	0.0017 (9)
C16	0.0560 (9)	0.0571 (10)	0.0980 (14)	−0.0107 (8)	0.0109 (9)	−0.0075 (9)
C17	0.0512 (7)	0.0313 (6)	0.0425 (7)	0.0004 (5)	0.0026 (6)	−0.0010 (5)
C18	0.0481 (7)	0.0369 (6)	0.0416 (7)	0.0041 (5)	−0.0002 (5)	0.0023 (5)
C19	0.0535 (8)	0.0378 (7)	0.0508 (8)	0.0004 (6)	0.0034 (6)	−0.0028 (6)
C20	0.0506 (8)	0.0504 (8)	0.0570 (9)	−0.0030 (6)	0.0012 (6)	0.0018 (7)
C21	0.0483 (8)	0.0636 (10)	0.0640 (10)	0.0033 (7)	0.0114 (7)	−0.0005 (8)
C22	0.0562 (9)	0.0530 (9)	0.0592 (9)	0.0098 (7)	0.0069 (7)	−0.0067 (7)
C23	0.0544 (8)	0.0381 (7)	0.0549 (8)	0.0028 (6)	0.0025 (6)	−0.0028 (6)
C24	0.0652 (10)	0.0628 (11)	0.0964 (14)	−0.0170 (8)	0.0124 (10)	−0.0043 (10)
C25	0.0850 (13)	0.0685 (12)	0.1098 (16)	0.0131 (10)	0.0317 (12)	−0.0227 (11)
N1	0.0548 (7)	0.0272 (5)	0.0495 (7)	0.0011 (5)	0.0016 (5)	0.0022 (5)
N2	0.0496 (6)	0.0315 (5)	0.0539 (7)	0.0017 (5)	0.0079 (5)	0.0049 (5)
O2	0.1092 (10)	0.0515 (6)	0.0522 (6)	0.0076 (6)	0.0257 (6)	0.0124 (5)
O3	0.1024 (9)	0.0384 (6)	0.0610 (7)	0.0002 (6)	−0.0009 (6)	−0.0085 (5)
O4	0.0645 (6)	0.0345 (5)	0.0598 (6)	0.0032 (4)	0.0096 (5)	0.0099 (4)

### Geometric parameters (Å, °)

**Table:**

C3—C2	1.423 (4)	C11—H11	0.9300
C3—O2	1.4416 (19)	C12—O3	1.2165 (17)
C3—C6'	1.464 (5)	C12—N1	1.3547 (19)
C3—C6	1.504 (4)	C13—N2	1.5143 (18)
C3—C2'	1.509 (4)	C13—C15	1.524 (3)
C3—C7	1.602 (4)	C13—C16	1.528 (2)
C3—C7'	1.629 (5)	C13—C14	1.532 (2)
C4—C5	1.390 (2)	C14—H14A	0.9600
C4—C8	1.3928 (19)	C14—H14B	0.9600
C4—C1	1.492 (11)	C14—H14C	0.9600
C4—C1'	1.492 (15)	C15—H15A	0.9600
C5—O2	1.3562 (17)	C15—H15B	0.9600
C5—C11	1.388 (2)	C15—H15C	0.9600
C1—O1	1.207 (16)	C16—H16A	0.9600
C1—C2	1.509 (12)	C16—H16B	0.9600
C2—H2A	0.9700	C16—H16C	0.9600
C2—H2B	0.9700	C17—O4	1.2310 (15)
C6—H6A	0.9600	C17—N2	1.3572 (17)
C6—H6B	0.9600	C17—C18	1.5016 (19)
C6—H6C	0.9600	C18—C19	1.3896 (19)
C7—H7A	0.9600	C18—C23	1.3898 (19)
C7—H7B	0.9600	C19—C20	1.387 (2)
C7—H7C	0.9600	C19—H19	0.9300
C1'—O1'	1.186 (19)	C20—C21	1.386 (2)
C1'—C2'	1.495 (15)	C20—C24	1.509 (2)
C2'—H2'1	0.9700	C21—C22	1.379 (2)
C2'—H2'2	0.9700	C21—H21	0.9300
C7'—H7'1	0.9600	C22—C23	1.382 (2)
C7'—H7'2	0.9600	C22—C25	1.512 (2)
C7'—H7'3	0.9600	C23—H23	0.9300
C6'—H6'1	0.9600	C24—H24A	0.9600
C6'—H6'2	0.9600	C24—H24B	0.9600
C6'—H6'3	0.9600	C24—H24C	0.9600
C8—C9	1.3794 (19)	C25—H25A	0.9600
C8—H8	0.9300	C25—H25B	0.9600
C9—C10	1.392 (2)	C25—H25C	0.9600
C9—C12	1.4962 (18)	N1—N2	1.3924 (15)
C10—C11	1.372 (2)	N1—H1	0.862 (17)
C10—H10	0.9300		
			
C2—C3—O2	116.33 (16)	C9—C10—H10	119.3
C2—C3—C6'	129.6 (3)	C10—C11—C5	120.18 (13)
O2—C3—C6'	113.1 (3)	C10—C11—H11	119.9
C2—C3—C6	116.2 (3)	C5—C11—H11	119.9
O2—C3—C6	107.3 (2)	O3—C12—N1	122.71 (13)
C2—C3—C2'	48.4 (2)	O3—C12—C9	122.21 (13)
O2—C3—C2'	116.81 (19)	N1—C12—C9	115.07 (11)
C6'—C3—C2'	114.3 (3)	N2—C13—C15	108.69 (13)
C6—C3—C2'	135.4 (3)	N2—C13—C16	109.02 (12)
C2—C3—C7	111.2 (3)	C15—C13—C16	112.19 (14)
O2—C3—C7	96.03 (19)	N2—C13—C14	108.80 (12)
C6'—C3—C7	72.1 (4)	C15—C13—C14	108.68 (15)
C6—C3—C7	107.7 (3)	C16—C13—C14	109.40 (15)
C2'—C3—C7	62.9 (3)	C13—C14—H14A	109.5
C2—C3—C7'	57.5 (2)	C13—C14—H14B	109.5
O2—C3—C7'	96.6 (2)	H14A—C14—H14B	109.5
C6'—C3—C7'	108.0 (4)	C13—C14—H14C	109.5
C6—C3—C7'	73.9 (3)	H14A—C14—H14C	109.5
C2'—C3—C7'	105.9 (3)	H14B—C14—H14C	109.5
C7—C3—C7'	166.1 (3)	C13—C15—H15A	109.5
C5—C4—C8	119.40 (13)	C13—C15—H15B	109.5
C5—C4—C1	119.3 (5)	H15A—C15—H15B	109.5
C8—C4—C1	120.5 (5)	C13—C15—H15C	109.5
C5—C4—C1'	119.5 (6)	H15A—C15—H15C	109.5
C8—C4—C1'	119.8 (6)	H15B—C15—H15C	109.5
O2—C5—C11	117.17 (13)	C13—C16—H16A	109.5
O2—C5—C4	123.34 (13)	C13—C16—H16B	109.5
C11—C5—C4	119.48 (13)	H16A—C16—H16B	109.5
O1—C1—C4	120.5 (10)	C13—C16—H16C	109.5
O1—C1—C2	125.0 (10)	H16A—C16—H16C	109.5
C4—C1—C2	114.3 (8)	H16B—C16—H16C	109.5
C3—C2—C1	115.7 (4)	O4—C17—N2	120.62 (12)
C3—C2—H2A	108.4	O4—C17—C18	119.25 (12)
C1—C2—H2A	108.4	N2—C17—C18	120.08 (11)
C3—C2—H2B	108.4	C19—C18—C23	119.26 (13)
C1—C2—H2B	108.4	C19—C18—C17	125.24 (12)
H2A—C2—H2B	107.4	C23—C18—C17	115.41 (12)
C3—C6—H6A	109.5	C20—C19—C18	120.35 (13)
C3—C6—H6B	109.5	C20—C19—H19	119.8
C3—C6—H6C	109.5	C18—C19—H19	119.8
C3—C7—H7A	109.5	C21—C20—C19	118.65 (14)
C3—C7—H7B	109.5	C21—C20—C24	119.92 (15)
C3—C7—H7C	109.5	C19—C20—C24	121.42 (14)
O1'—C1'—C4	125.0 (12)	C22—C21—C20	122.29 (14)
O1'—C1'—C2'	118.7 (12)	C22—C21—H21	118.9
C4—C1'—C2'	116.2 (10)	C20—C21—H21	118.9
C1'—C2'—C3	112.6 (6)	C21—C22—C23	118.06 (14)
C1'—C2'—H2'1	109.1	C21—C22—C25	120.98 (15)
C3—C2'—H2'1	109.1	C23—C22—C25	120.94 (15)
C1'—C2'—H2'2	109.1	C22—C23—C18	121.33 (13)
C3—C2'—H2'2	109.1	C22—C23—H23	119.3
H2'1—C2'—H2'2	107.8	C18—C23—H23	119.3
C3—C7'—H7'1	109.5	C20—C24—H24A	109.5
C3—C7'—H7'2	109.5	C20—C24—H24B	109.5
H7'1—C7'—H7'2	109.5	H24A—C24—H24B	109.5
C3—C7'—H7'3	109.5	C20—C24—H24C	109.5
H7'1—C7'—H7'3	109.5	H24A—C24—H24C	109.5
H7'2—C7'—H7'3	109.5	H24B—C24—H24C	109.5
C3—C6'—H6'1	109.5	C22—C25—H25A	109.5
C3—C6'—H6'2	109.5	C22—C25—H25B	109.5
H6'1—C6'—H6'2	109.5	H25A—C25—H25B	109.5
C3—C6'—H6'3	109.5	C22—C25—H25C	109.5
H6'1—C6'—H6'3	109.5	H25A—C25—H25C	109.5
H6'2—C6'—H6'3	109.5	H25B—C25—H25C	109.5
C9—C8—C4	121.44 (13)	C12—N1—N2	120.89 (11)
C9—C8—H8	119.3	C12—N1—H1	123.0 (11)
C4—C8—H8	119.3	N2—N1—H1	115.2 (11)
C8—C9—C10	118.11 (12)	C17—N2—N1	119.06 (11)
C8—C9—C12	123.42 (12)	C17—N2—C13	122.53 (11)
C10—C9—C12	118.41 (12)	N1—N2—C13	117.73 (10)
C11—C10—C9	121.39 (13)	C5—O2—C3	119.03 (12)
C11—C10—H10	119.3		
			
C8—C4—C5—O2	178.51 (15)	C4—C5—C11—C10	1.2 (2)
C1—C4—C5—O2	8.4 (4)	C8—C9—C12—O3	159.80 (15)
C1'—C4—C5—O2	−14.6 (4)	C10—C9—C12—O3	−17.1 (2)
C8—C4—C5—C11	−0.8 (2)	C8—C9—C12—N1	−20.8 (2)
C1—C4—C5—C11	−170.9 (4)	C10—C9—C12—N1	162.30 (13)
C1'—C4—C5—C11	166.0 (4)	O4—C17—C18—C19	−134.18 (14)
C5—C4—C1—O1	179.1 (7)	N2—C17—C18—C19	48.51 (19)
C8—C4—C1—O1	9.2 (9)	O4—C17—C18—C23	42.43 (18)
C1'—C4—C1—O1	−85 (3)	N2—C17—C18—C23	−134.88 (13)
C5—C4—C1—C2	5.0 (7)	C23—C18—C19—C20	1.9 (2)
C8—C4—C1—C2	−164.9 (4)	C17—C18—C19—C20	178.41 (13)
C1'—C4—C1—C2	101 (3)	C18—C19—C20—C21	0.0 (2)
O2—C3—C2—C1	41.7 (5)	C18—C19—C20—C24	−179.00 (15)
C6'—C3—C2—C1	−150.7 (6)	C19—C20—C21—C22	−1.9 (2)
C6—C3—C2—C1	169.6 (5)	C24—C20—C21—C22	177.18 (17)
C2'—C3—C2—C1	−61.8 (5)	C20—C21—C22—C23	1.7 (2)
C7—C3—C2—C1	−66.8 (5)	C20—C21—C22—C25	−177.15 (17)
C7'—C3—C2—C1	122.4 (5)	C21—C22—C23—C18	0.4 (2)
O1—C1—C2—C3	156.6 (8)	C25—C22—C23—C18	179.19 (16)
C4—C1—C2—C3	−29.6 (7)	C19—C18—C23—C22	−2.1 (2)
C5—C4—C1'—O1'	177.5 (8)	C17—C18—C23—C22	−178.97 (13)
C8—C4—C1'—O1'	−15.7 (11)	O3—C12—N1—N2	−11.2 (2)
C1—C4—C1'—O1'	82 (3)	C9—C12—N1—N2	169.32 (11)
C5—C4—C1'—C2'	−3.3 (8)	O4—C17—N2—N1	−175.61 (12)
C8—C4—C1'—C2'	163.5 (4)	C18—C17—N2—N1	1.66 (18)
C1—C4—C1'—C2'	−99 (3)	O4—C17—N2—C13	−5.3 (2)
O1'—C1'—C2'—C3	−151.4 (8)	C18—C17—N2—C13	171.97 (12)
C4—C1'—C2'—C3	29.4 (8)	C12—N1—N2—C17	84.40 (16)
C2—C3—C2'—C1'	61.4 (5)	C12—N1—N2—C13	−86.37 (16)
O2—C3—C2'—C1'	−41.0 (6)	C15—C13—N2—C17	61.36 (17)
C6'—C3—C2'—C1'	−176.2 (6)	C16—C13—N2—C17	−61.21 (18)
C6—C3—C2'—C1'	148.0 (6)	C14—C13—N2—C17	179.54 (14)
C7—C3—C2'—C1'	−123.8 (6)	C15—C13—N2—N1	−128.22 (13)
C7'—C3—C2'—C1'	65.1 (6)	C16—C13—N2—N1	109.21 (14)
C5—C4—C8—C9	0.1 (2)	C14—C13—N2—N1	−10.03 (19)
C1—C4—C8—C9	170.0 (4)	C11—C5—O2—C3	−177.56 (14)
C1'—C4—C8—C9	−166.8 (4)	C4—C5—O2—C3	3.1 (2)
C4—C8—C9—C10	0.3 (2)	C2—C3—O2—C5	−28.9 (3)
C4—C8—C9—C12	−176.65 (14)	C6'—C3—O2—C5	161.4 (4)
C8—C9—C10—C11	0.1 (2)	C6—C3—O2—C5	−160.9 (3)
C12—C9—C10—C11	177.22 (13)	C2'—C3—O2—C5	25.7 (3)
C9—C10—C11—C5	−0.9 (2)	C7—C3—O2—C5	88.4 (2)
O2—C5—C11—C10	−178.14 (14)	C7'—C3—O2—C5	−85.8 (2)

### Hydrogen-bond geometry (Å, °)

**Table:**

*D*—H···*A*	*D*—H	H···*A*	*D*···*A*	*D*—H···*A*
C16—H16C···O4	0.96	2.39	3.007 (2)	121
C15—H15A···O4	0.96	2.36	2.918 (2)	117
C8—H8···O4^i^	0.93	2.48	3.3214 (18)	151
N1—H1···O4^i^	0.862 (17)	2.096 (18)	2.9442 (15)	167.8 (15)

Symmetry codes: (i) −*x*+3/2, *y*+1/2, −*z*+1/2.
